# Supplementary figures and images for: Massive colonization of protein-coding exons by selfish genetic elements in Paramecium germline genomes
Source: PLoS Biol. 2021 Jul 29;19(7):e3001309. doi: 10.1371/journal.pbio.3001309 (PMC8354472; doi:10.1371/journal.pbio.3001309)

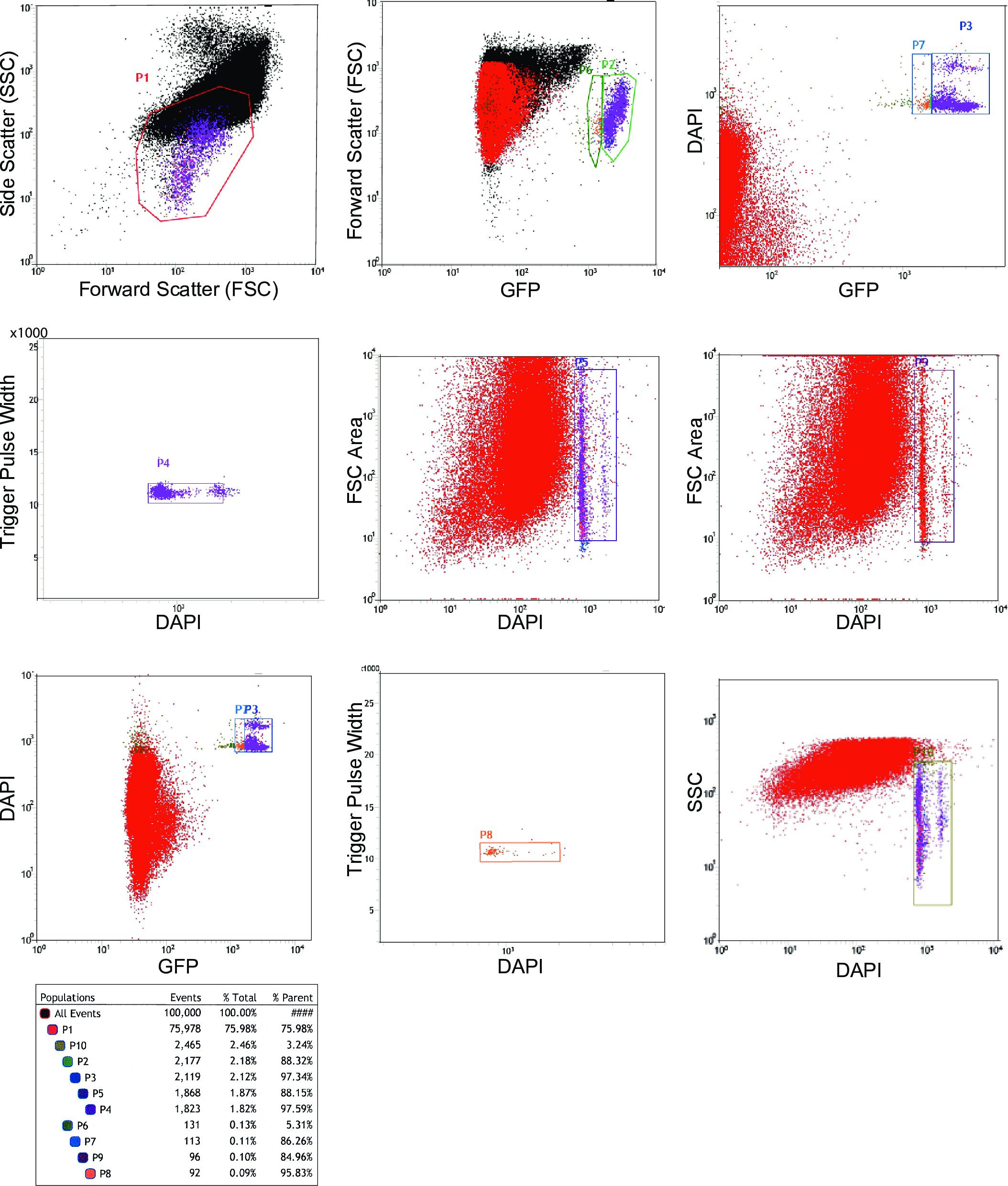

Supplement: S1 Fig — GFP, DAPI-positive MICs from P. sonneborni vegetative cells transformed with the P. tetraurelia CENH3a-GFP transgene [62] were sorted based on size, granularity, DAPI staining, and GFP signal (see Materials and methods). P4 and P8 were sorted separately. Based on quality control by flow imaging (Imagestream) indicating 97% purity, the 2 samples P4 and P8, which represent 1.91% of total events, were combined for DNA extraction and sequencing. Two populations are visualized and likely correspond to 2n and 4n MICs. FSC, forward scatter; GFP, green fluorescent protein; MIC, micronucleus; SSC, side scatter. (TIF) [file pbio.3001309.s001.tif]

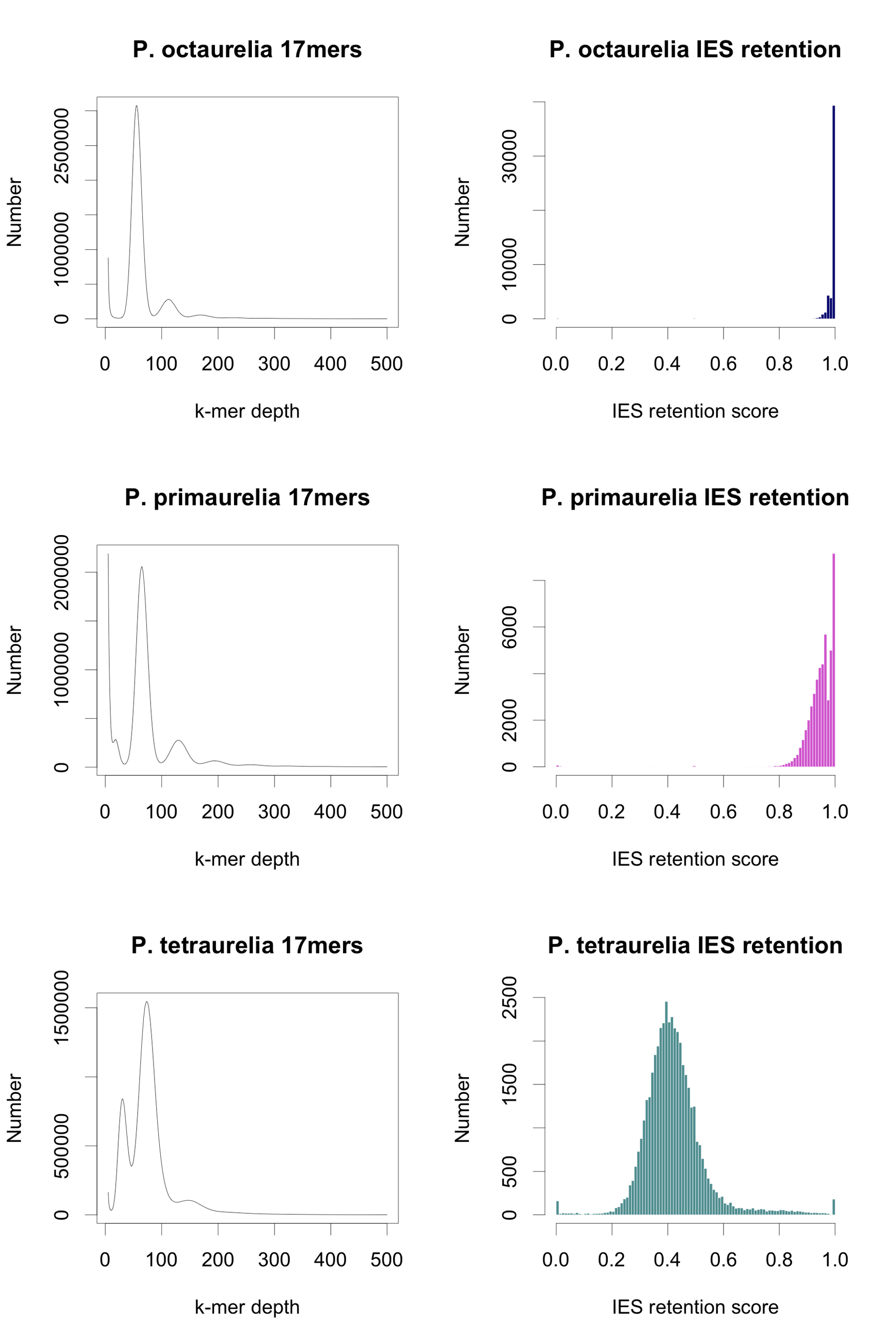

Supplement: S2 Fig — The histograms on the left show the k-mer depth profiles. The peak at the origin can be attributed to sequencing errors (k-mers that occur only once or a few times). The position of the largest peak beyond the origin corresponds to k-mers present once in the genome and provides the sequencing depth. As P. aurelia genomes have undergone whole genome duplications, there are a significant number of k-mers at 2X and even 4X the sequencing depth arising from genes (or regions of genes) present in 2 or 4 copies, clearly visible for P. octaurelia and P. primaurelia. The profile for P. tetraurelia, however, has a first peak (MIC sequences that occur once) at 31X followed by a larger peak that is not at the 2X position as it arises because of MAC DNA contamination. The column on the right shows histograms of IRS. Only the P. tetraurelia sample is significantly contaminated by MAC DNA: The average IRS of 0.4 indicates 40% MIC and 60% MAC DNA in this sample. The data underlying this figure may be found at https://doi.org/10.5281/zenodo.4836464. IES, internal eliminated sequence; IRS, IES retention score; MAC, macronucleus; MIC, micronucleus. (TIF) [file pbio.3001309.s002.tif]

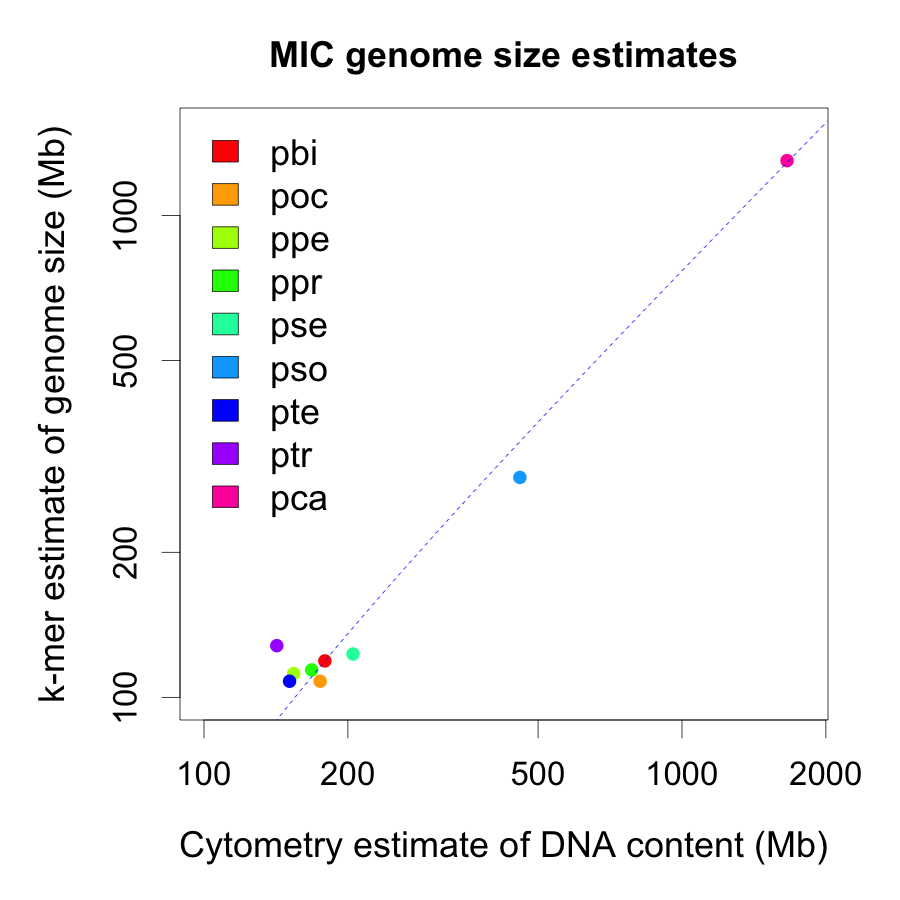

Supplement: S3 Fig — Flow cytometry estimates of DNA content of micronuclei and k-mer counting estimates of genome size are described in Materials and methods. In order to show all of the data, both axes of the graph are log transformed. Simple linear regression was carried out on the untransformed data with R. The linear model that fits the data is presented as a dashed blue line; R2 = 0.99, p-value = 1.3 × 10^−09^. The data underlying this figure may be found at https://doi.org/10.5281/zenodo.4836464. MIC, micronucleus; pbi, P. biaurelia; pca, P. caudatum; poc, P. octaurelia; ppe, P. pentaurelia; ppr, P. primaurelia; pso, P. sonneborni; pse, P. sexaurelia; pte, P. tetraurelia; ptr, P. tredecaurelia. (TIF) [file pbio.3001309.s003.tif]

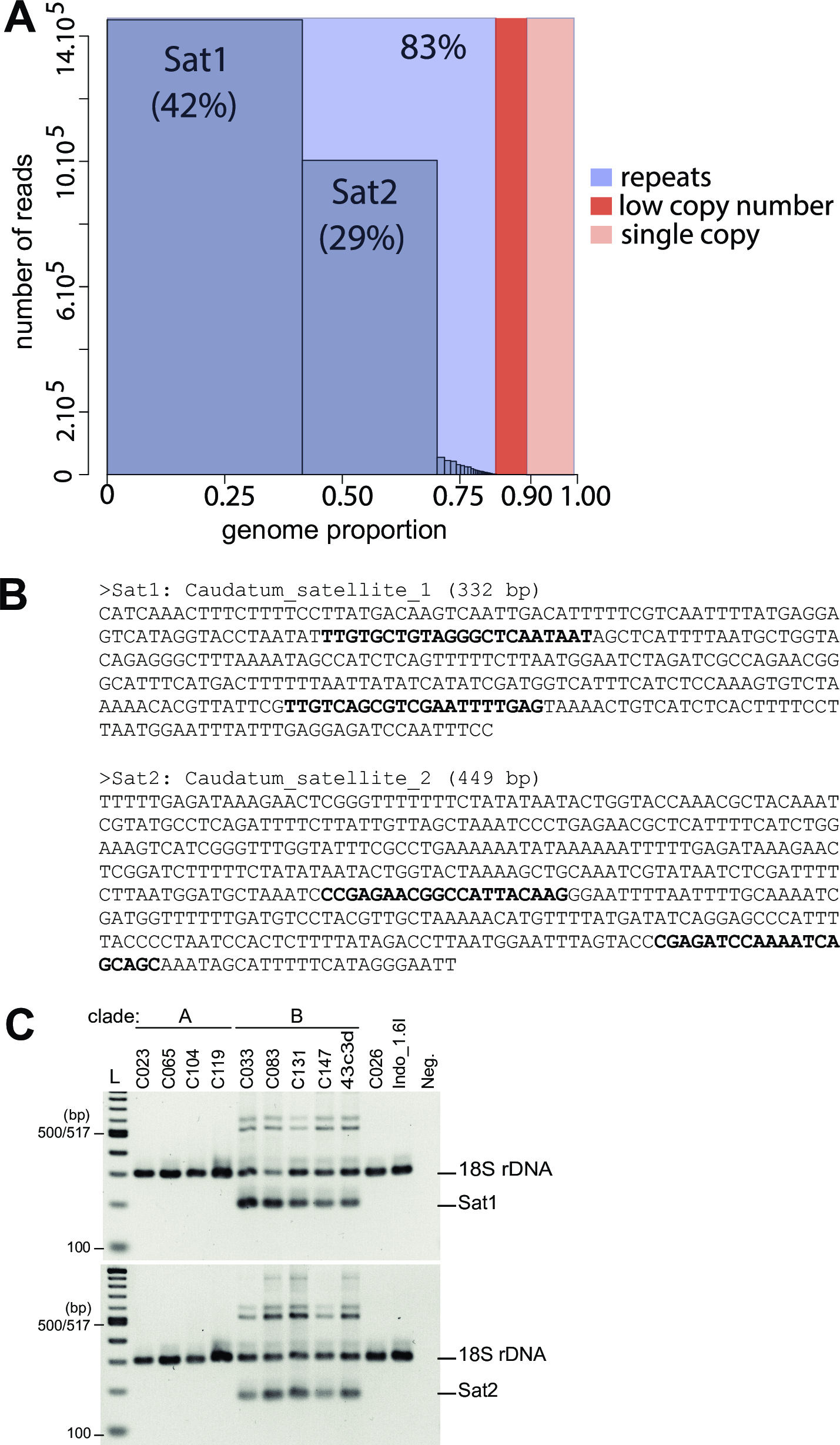

Supplement: S4 Fig — (A) Abundance of repeat families identified by DNAPipeTE in P. caudatum strain My43c3d. The repeat content of the P. caudatum MIC genome was analyzed with DNAPipeTE [86], using a sample of 3,500,000 sequence reads (corresponding to a read depth of approximately 0.5X). DNAPipeTE identified 67 repeat families that collectively constitute 83% of the MIC genome. Among them, there are 2 major satellite repeats Sat1 and Sat2, which represent, respectively, 42% and 29% of the MIC genome. The data underlying this panel may be found at https://doi.org/10.5281/zenodo.4836464. (B) Sequences of the 2 major satellite repeats Sat1 and Sat2 in P. caudataum My43c3d (332 bp and 449 bp long). These 2 satellite repeats share homology over an approximately 200-bp-long region. Primer sequences used for specific PCR amplification of each repeat are indicated in bold. (C) Detection of Sat1 and Sat2 in P. caudatum strains. Whole-cell genomic DNA was used to perform duplex PCR with a set of primers located within each repeat (Sat1 or Sat2, in bold panel B) and another set of primers within the 18S ribosomal DNA as a loading control. The expected size of the 18SrDNA PCR product was 301 bp using primers 18S_F953: AGACGATCAGATACCGTCGTAG and 18S_R1300: CACCAACTAAGAACGGCCATGC. L: 1-kb NEB ladder. Neg.: negative control (no DNA). Sat1 was amplified with primers comp2975_F1: TTGTGCTGTAGGGCTCAATAAT and comp2975_R1: CTCAAAATTCGACGCTGACAA at the expected size (198 bp) in the P. caudatum clade B strains tested (My43c3d; C033; C083; C131; C147). The repeat could not be amplified in P. caudatum DNA from clade A strains (C023; C065; C104; C119), from strain C026 or from strain Indo_1.6I. Sat2 was amplified with primers comp5240_F1: TGCTGCTGATTTTGGATCTCG and comp5240_R1: CCGAGAACGGCCATTACAAG at the expected size (168 bp) in the P. caudatum clade B strains tested (My43c3d; C033; C083; C131; C147). The repeat could not be amplified in P. caudatum DNA from clade A strains (C023; C065; C104; C119), from strai [file pbio.3001309.s004.tif]

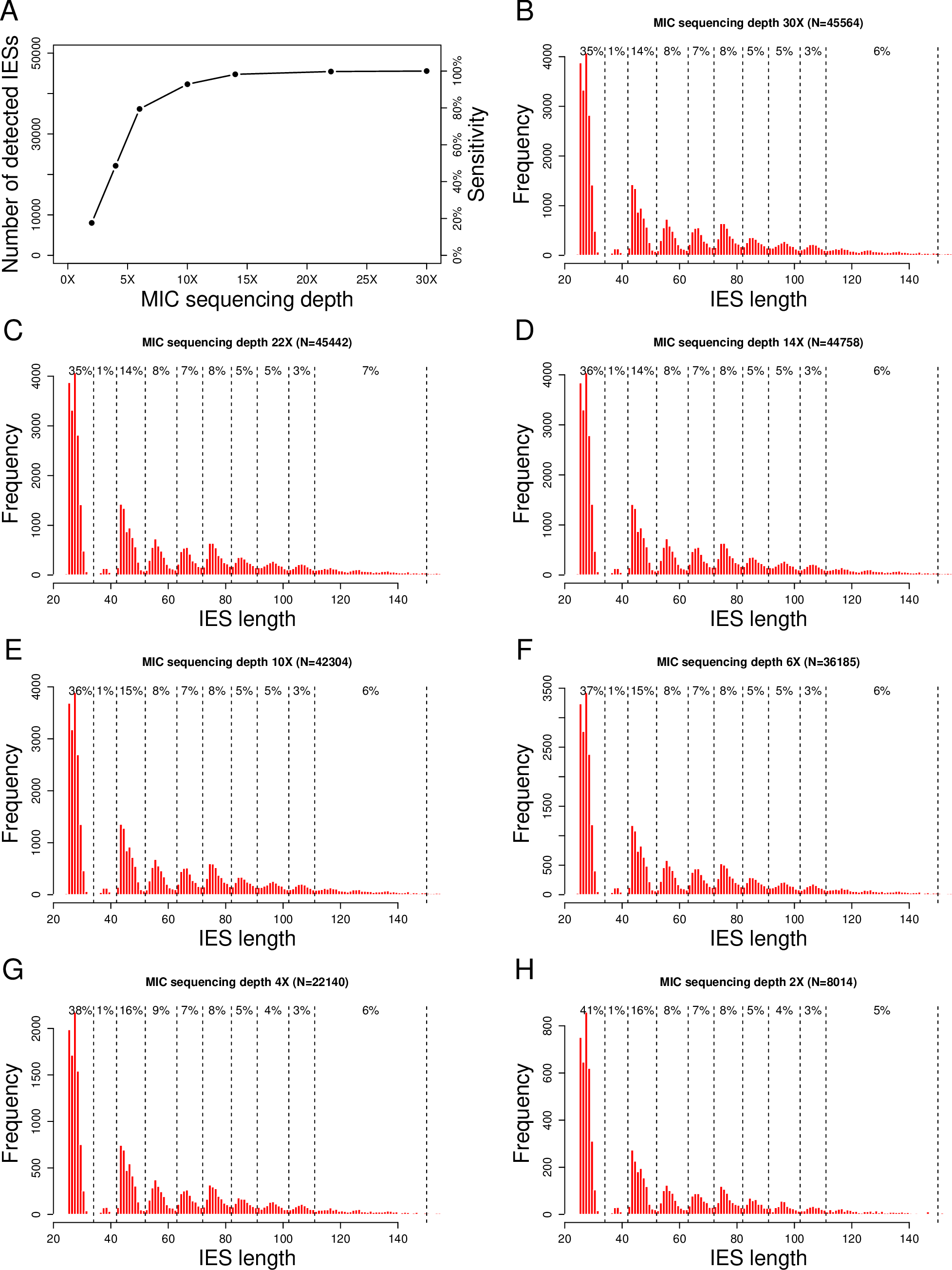

Supplement: S5 Fig — To assess the impact of sequencing depth on the sensitivity of IES detection, we subsampled sequence reads from the P. tetraurelia MIC dataset so as to obtain subsets of lower depth (from 2× to 30×), on which we applied the same IES detection procedure. (A) Number of detected IESs vs. sequencing depth. (B–H) Length distributions of IESs detected within each subset (the percentage of IESs in each peak is indicated). Sequencing depth affects the number of detected IESs (for depths <15×), but not their length distribution. The data underlying this figure may be found at https://doi.org/10.5281/zenodo.4836464. IES, internal eliminated sequence; MIC, micronucleus. (TIF) [file pbio.3001309.s005.tif]

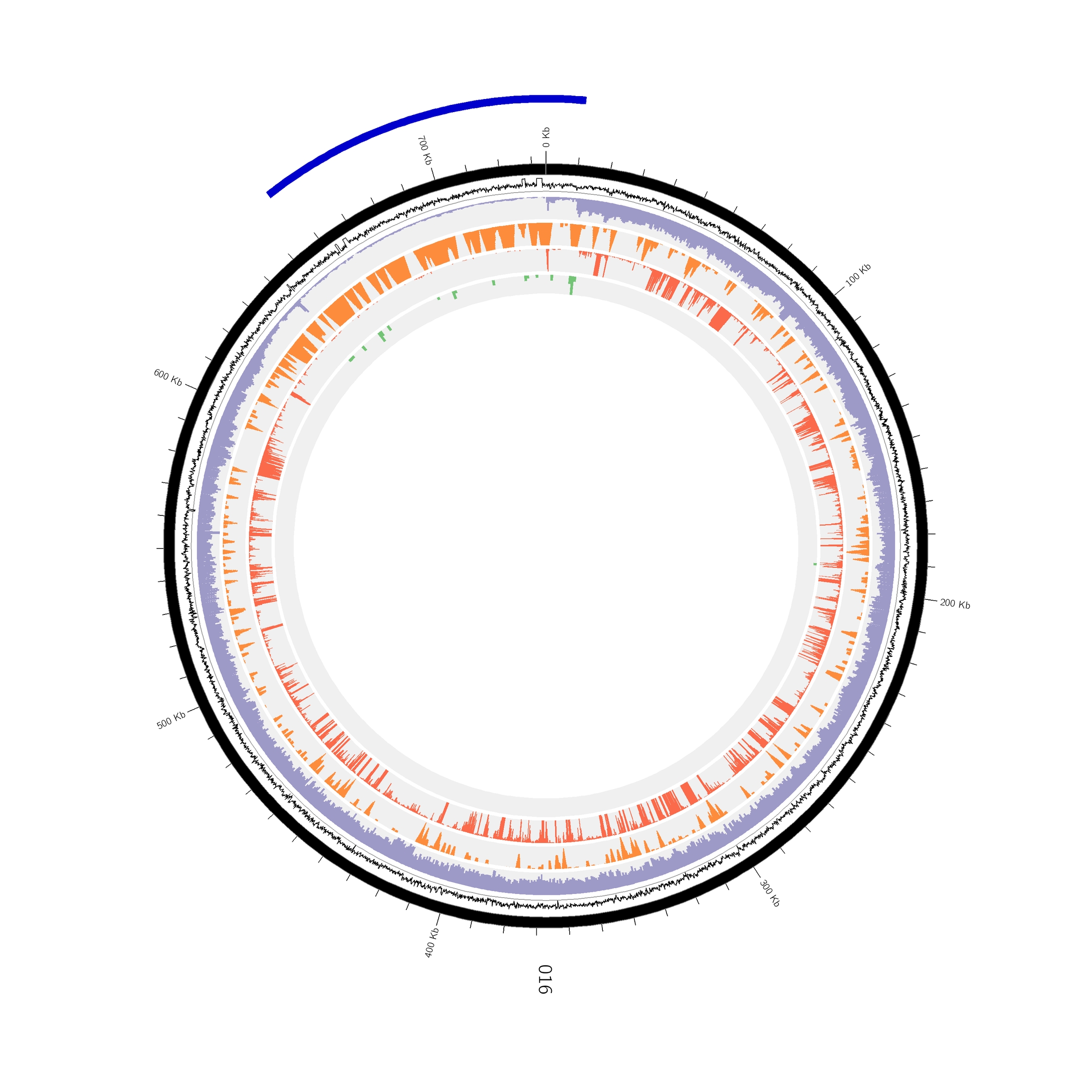

Supplement: S6 Fig — Circular representation of 1 scaffold of approximately 730 kb. The tracks from the exterior to the interior of the circle: G+C content of 100 nt sliding windows (black), MAC DNA-seq depth (purple), the density in predicted noncoding genes (orange), RNA-Seq depth (red), and the density of detected telomerisation sites (green). The external blue arc shows the region identified as being MAC variable. These regions were determined by an automatic pipeline (see Materials and methods), then adjusted by eye for each scaffold. MAC, macronucleus. (TIF) [file pbio.3001309.s006.tif]

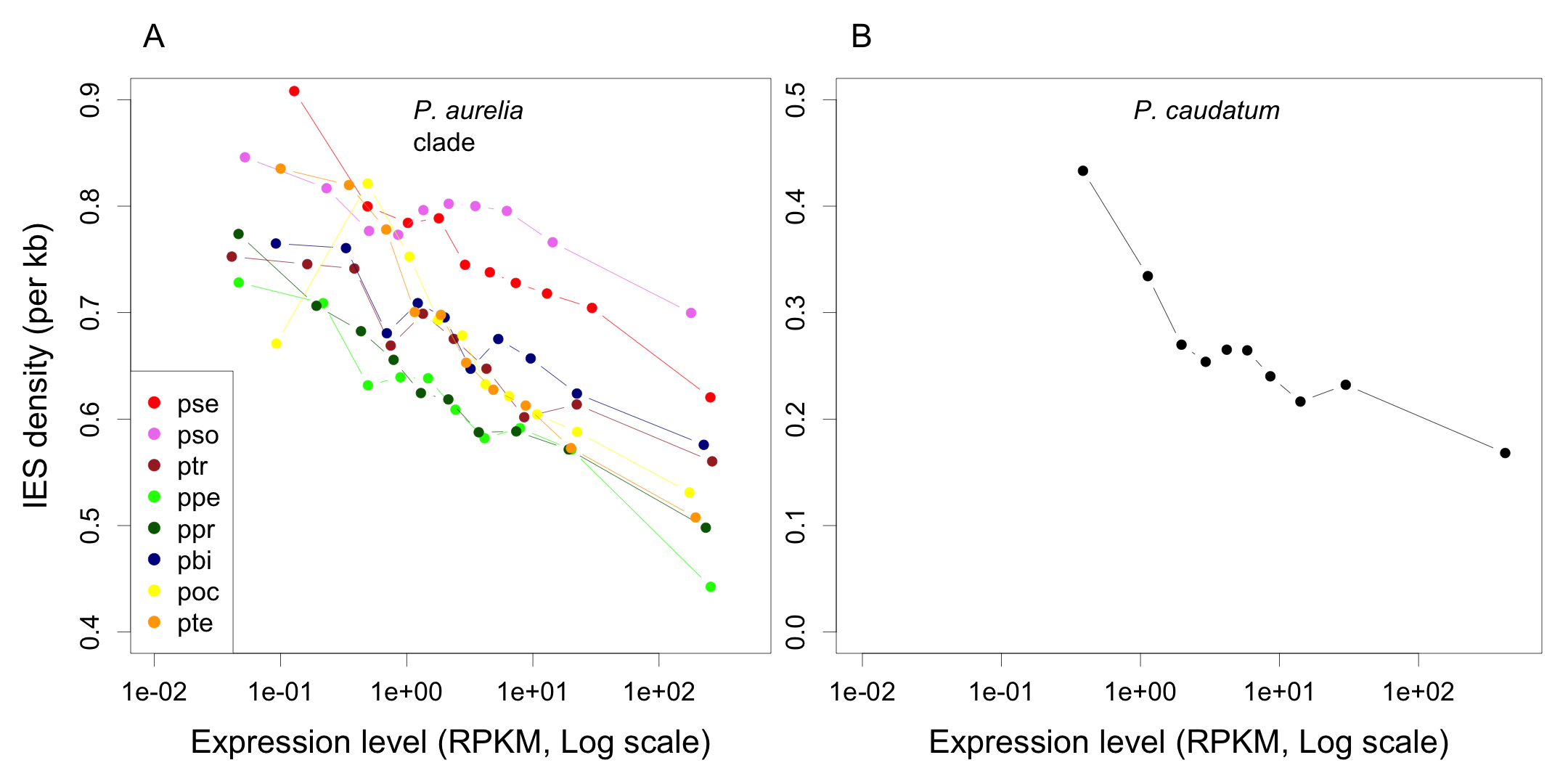

Supplement: S7 Fig — Expression levels (RPKM) were measured with RNA-Seq datasets from vegetative cells. For each species, expressed genes were classified into 10 bins of equal sample size according to their expression level, and IES density computed within each bin. Nonexpressed genes (6.6% of the entire dataset) were excluded. (A) P. aurelia species. (B) P. caudatum. The data underlying this figure may be found at https://doi.org/10.5281/zenodo.4836464. IES, internal eliminated sequence; pbi, P. biaurelia; pca, P. caudatum; poc, P. octaurelia; ppe, P. pentaurelia; ppr, P. primaurelia; pso, P. sonneborni; pse, P. sexaurelia; pte, P. tetraurelia; ptr, P. tredecaurelia. (TIF) [file pbio.3001309.s007.tif]

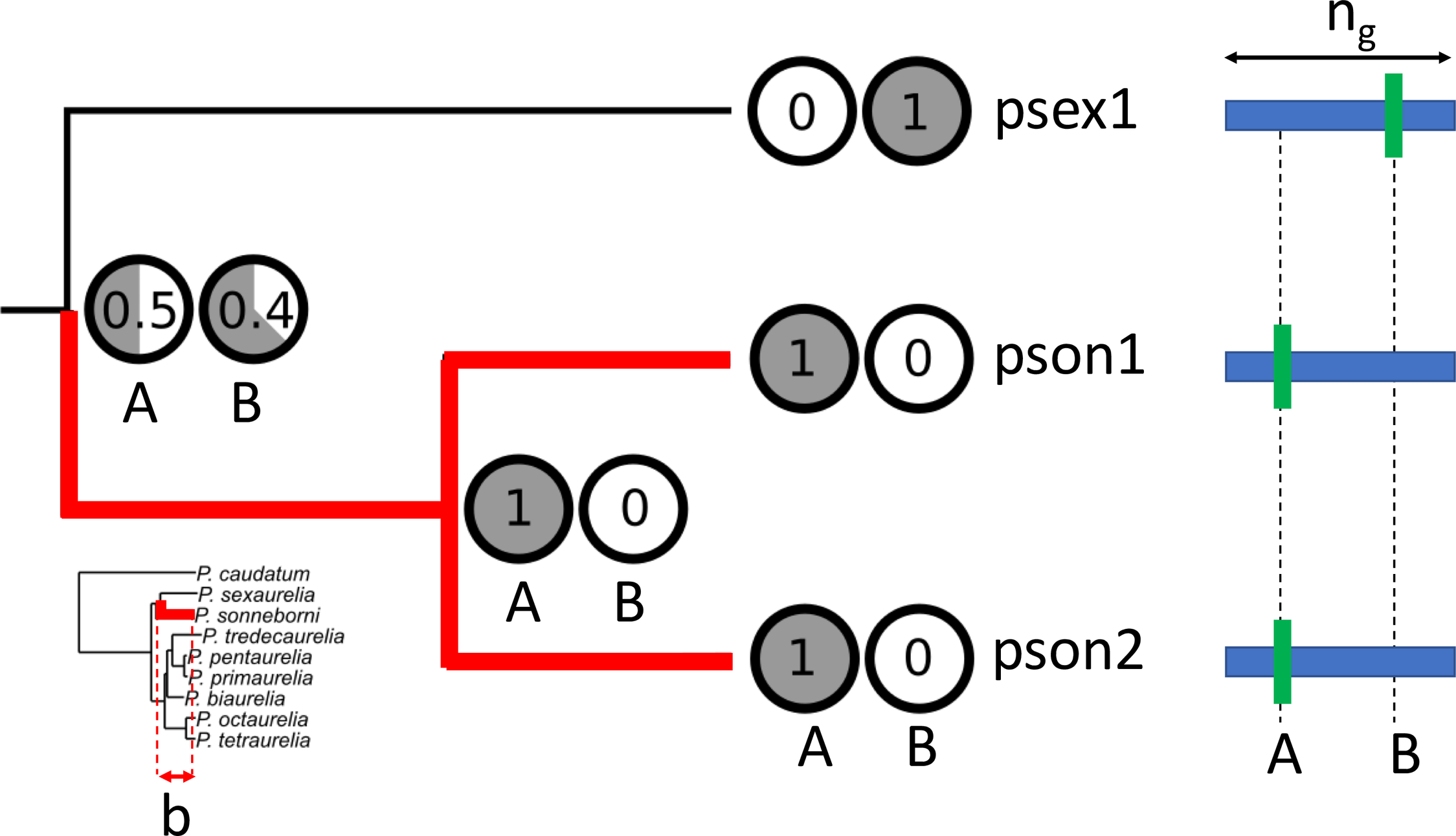

Supplement: S8 Fig — To illustrate our methodology, we show here an example of a gene family with 3 genes, 2 from P. sonneborni (pson1, pson2) and 1 from P. sexaurelia (psex1). Two IES loci are found in this family (A, B). The probability of presence of an IES (estimated by Bayesian ancestral state reconstruction—see Materials and methods) is indicated by shaded circles for each locus at each node of the gene phylogeny. We focus here on the branch of the species tree leading from the common ancestor of P. sexaurelia and P. sonneborni to the leaf node of P. sonneborni (the red branch in the species tree, shown in the insert). The length of this branch (b) is taken as a proxy for time. Because of a duplication event, this branch of the species tree corresponds to 2 paths in the gene tree (k = 2). To estimate the IES gain rate, we calculate for each path the sum of increase in the probability of presence of an IES, for all IES loci (p+). Along the first path (from the root to pson1), we have p+A1 = 0.5 and p+B1 = 0. Along the second path (from the root to pson2), we have p+A2 = 0.5 and p+B2 = 0. The average gain rate along all paths, per unit of time and per bp, is thus given by G = (p+A1 + p+B1 + p+A2 + p+B2) / (k × b × ng), where ng is the number of well-aligned sites in the gene family alignment (i.e., the number of sites where the presence of co-orthologous IESs can be assessed). Similarly, to estimate the IES loss rate, we calculate for each path the sum of decrease in the probability of presence of an IES, for all IES loci (p−). Along the first path (from the root to pson1), we have p−A1 = 0 and p−B1 = 0.4. Along the second path (from the root to pson2), we have p−A2 = 0 and p−B2 = 0.4. The average gain rate along all paths, per unit of time and per bp, is thus given by L = (p−A1 + p−B1 + p−A2 + p−B2) / (k × b × I), where I is the number of IES loci in the gene family (here I = 2). IES, internal eliminated sequence. (TIF) [file pbio.3001309.s008.tif]

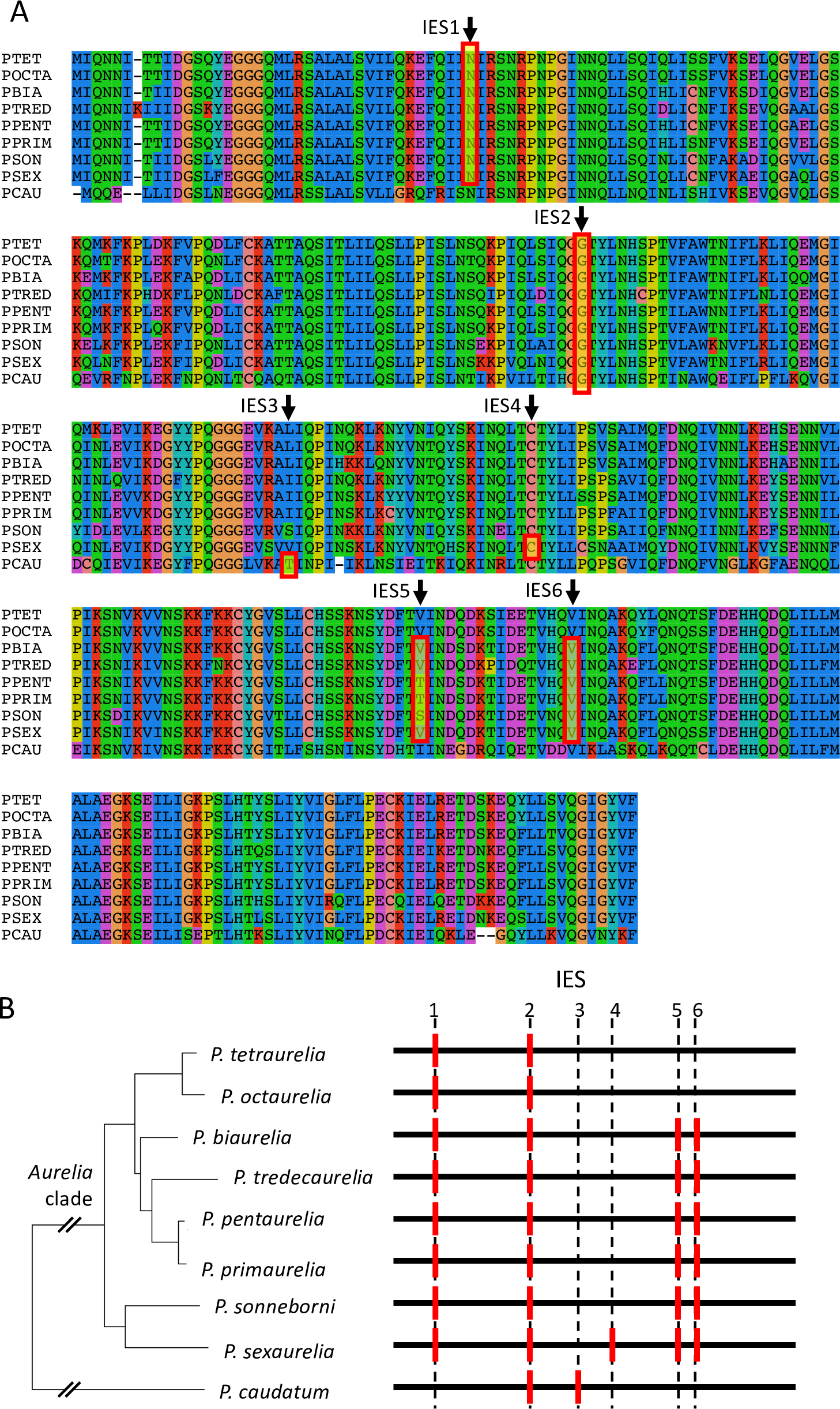

Supplement: S9 Fig — (A) To date events of IES loss or gain, it is first necessary to identify IESs that derive from a single ancestral insertion event (co-orthologous IESs). For this, we aligned coding sequences (based on the protein alignment) and mapped the position of IESs: IESs located at the exact same position within a codon were assumed to be co-orthologous. We then used the reconciled gene tree to map events in the species phylogeny, using a maximum likelihood approach (see Materials and methods). The example shown here corresponds to a gene family encoding a putative RNA 3′-terminal phosphate cyclase (PTET.51.1.P0920097, POCTA.138.1.P0960088, PBIA.V1_4.1.P01950012, PTRED.209.2.P71800001293600070, PPENT.87.1.P1090087, PPRIM.AZ9–3.1.P0020612, PSON.ATCC_30995.1.P0860097, PSEX.AZ8_4.1.P0910047, PCAU.43c3d.1.P00760109). The positions of IESs are indicated by red rectangles. (B) The presence of IESs (red bars) within each of these genes is indicated with regard to the species phylogeny. Six distinct IESs were identified in this gene family: IES2 is shared by all species and therefore predates the divergence between P. caudatum and the aurelia clade; IES4 most probably corresponds to a gain in the P. sexaurelia lineage; IES5 and IES6 predate the divergence of the aurelia clade and have been subsequently lost in the P. tetraurelia/P. octaurelia lineage; IES1 might correspond to a gain at the base of the aurelia clade or a loss in the P. caudatum lineage (and vice versa for IES3). IES, internal eliminated sequence. (TIF) [file pbio.3001309.s009.tif]

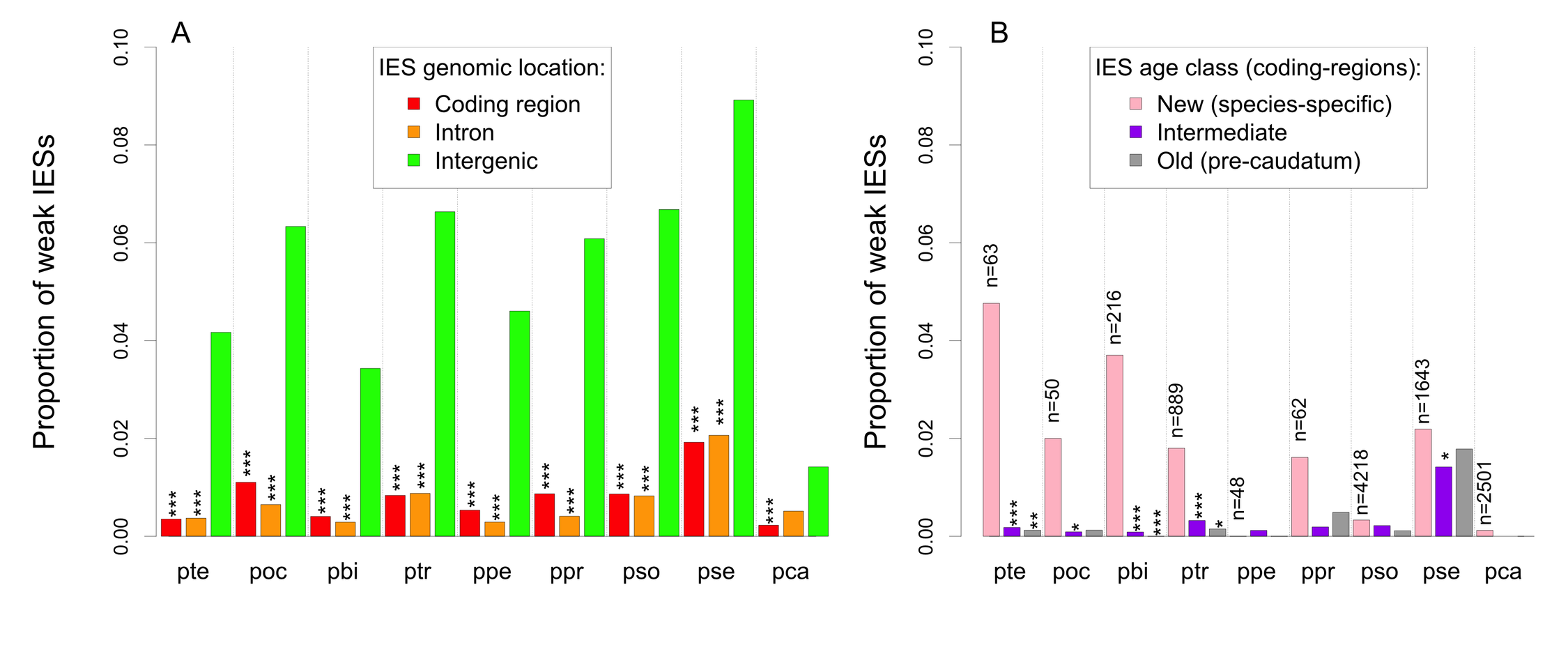

Supplement: S10 Fig — (A) Proportion of weak IESs (i.e., IESs with a retention frequency ≥10% in WT vegetative cells) among IESs located in different genomic compartments. The proportion of weak IESs among intergenic IESs was compared to that of IESs located in introns or coding regions, by a chi-squared test. (B) Proportion of weak IESs according to the age of IESs (for IESs located in coding regions): New = species-specific IES; Old = IES predating the divergence between P. caudatum and the aurelia lineage. The number of new IESs is indicated for each species. The proportion of weak IESs among new IESs was compared to that of older ones, by a chi-squared test. Species codes: pbi, P. biaurelia; pca, P. caudatum; poc, P. octaurelia; ppe, P. pentaurelia; ppr, P. primaurelia; pso, P. sonneborni; pse, P. sexaurelia; pte, P. tetraurelia; ptr, P. tredecaurelia. (*: p-value < 0.05; **: p-value < 1e-3; ***: p-value < 1e-6). The data underlying this figure may be found at https://doi.org/10.5281/zenodo.4836464. IES, internal eliminated sequence; WT, wild-type. (TIF) [file pbio.3001309.s010.tif]

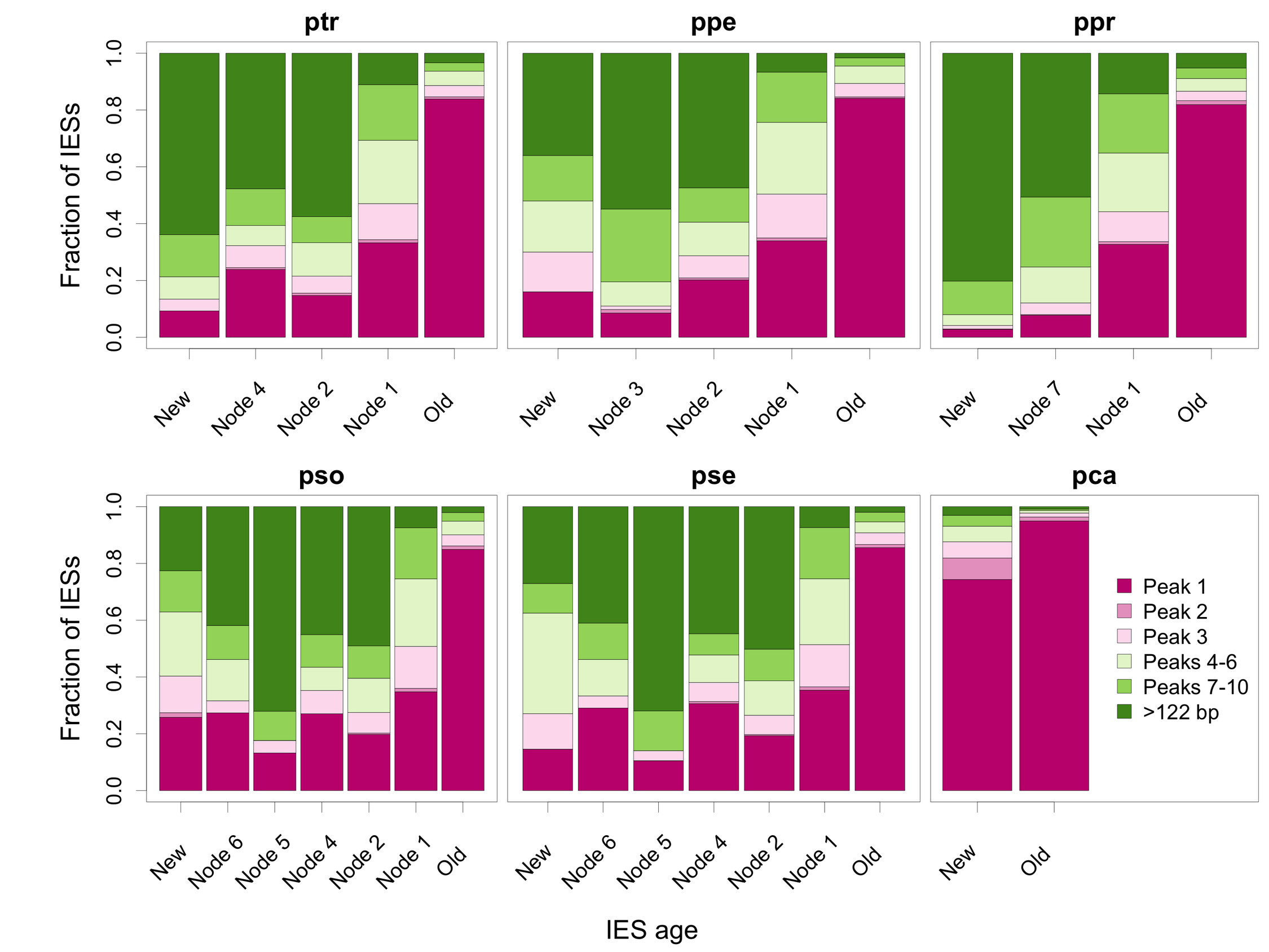

Supplement: S11 Fig — Comparison of the length distribution of IESs according to their age (for the subset of datable IESs located in coding regions). The age of an IES site is defined as in Fig 3. Results for other species are shown in Fig 4. The data underlying this figure may be found at https://doi.org/10.5281/zenodo.4836464. IES, internal eliminated sequence; pca, P. caudatum; ppe, P. pentaurelia; ppr, P. primaurelia; pso, P. sonneborni; pse, P. sexaurelia; ptr, P. tredecaurelia. (TIF) [file pbio.3001309.s011.tif]

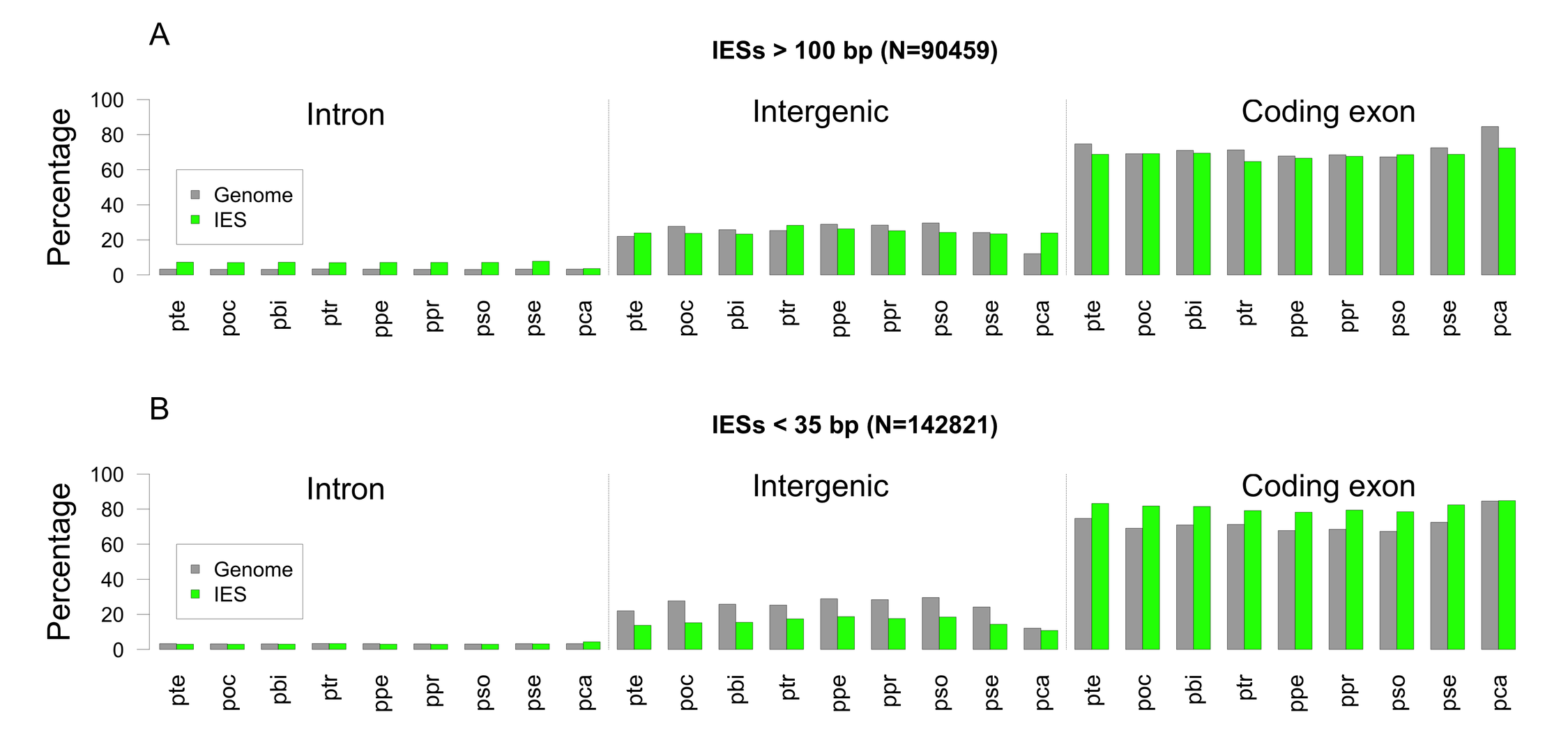

Supplement: S12 Fig — Green bars indicate the percentage of IESs located in each compartment of the MAC genome (introns, protein-coding regions, and intergenic regions) for each species. Gray bars indicate the percentage of the MAC genome in each compartment. (A) Long IESs (>100 bp). (B) Short IESs (<35 bp). For each species, the relative proportion of IESs in the 3 compartments (intron, intergenic, and coding regions) was compared for short IESs (<35 bp) vs long IESs (>100 bp) by a chi-squared test (p-value < 10−16 in all species). The data underlying this figure may be found at https://doi.org/10.5281/zenodo.4836464. IES, internal eliminated sequence; MAC, macronucleus. (TIF) [file pbio.3001309.s012.tif]

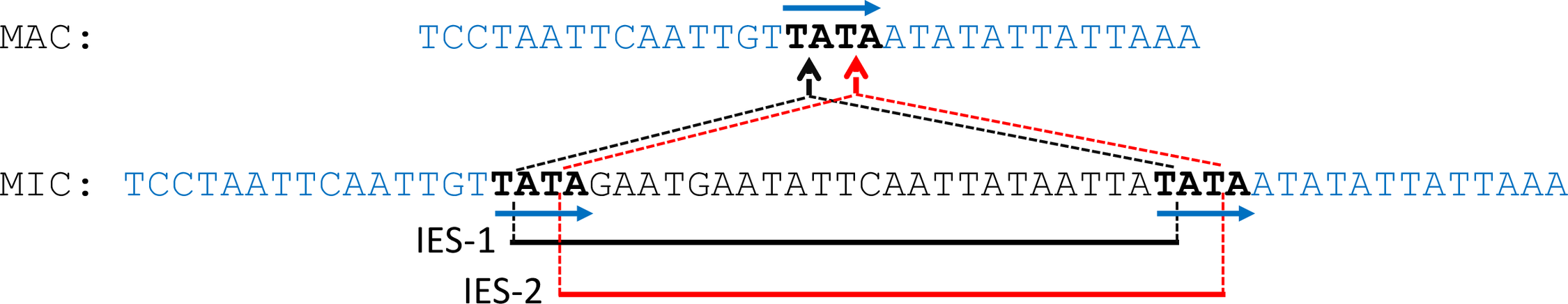

Supplement: S13 Fig — Comparison of MIC and MAC sequences indicates the presence of an IES at this locus. However, because of the presence of a repeated motif at the boundaries of the IES (blue arrows), it is not possible to determine which of the 2 possible segments (IES-1 in black or IES-2 in red) is actually excised in vivo. Such IESs that cannot be unambiguously positioned are called “floating IESs.” They represent 6.8% of the 400,254 IESs detected across all species. In the vast majority of cases (86%), the alternative locations of floating IESs differ by only 2 bp (as in the example shown here), and there are less than 1% of floating IESs for which the uncertainty in IES position exceeds 5 bp. IES, internal eliminated sequence; MAC, macronucleus; MIC, micronucleus. (TIF) [file pbio.3001309.s013.tif]
