# Supplementary material for: Massive colonization of protein-coding exons by selfish genetic elements in Paramecium germline genomes
Source: PLoS Biol. 2021 Jul 29;19(7):e3001309. doi: 10.1371/journal.pbio.3001309 (PMC8354472; doi:10.1371/journal.pbio.3001309)
Supplement: Attachment — Submitted filename: ResponseToReviewers_Final.docx [file pbio.3001309.s023.docx]

# Response to reviewers.

We thank all three reviewers for their constructive comments. In the text below, the reviewer’s comments are printed in blue, and our response in black. We have numbered the reviewers’ comments to facilitate referencing.

# Reviewer #1:

Using whole genome sequencing of the MIC and MAC genomes of nine Paramecium species, Sellis and Guérin present a convincing and highly significant demonstration of what has been suspected in the field for decades -- that the IES sequences in these species are born from transposable elements. It is hard to imagine any other way this elaborate and amazing state of the genome could arise, but the presumed solution has long lacked convincing empirical evidence. The key innovation here was the group's generation of whole genome sequences for diverse Paramecium species. In some cases the cross-species comparison of IES sequences may have been useful, but the limiting factor in previous attempts to solve this problem seems to have been that the model genome (P. tetraurelia) has few IESs with detectable repeat character, compared to some species with many more. The current study overcomes this so show that most species have many IESs derived from repeats and repeats that are likely

transposable elements. The rest of the paper analyzes several fascinating aspects of these genome sequence including a presumed horizontal transfer of TEs and co-option of some TE/IES sequences for host genome function. The major flaws in the paper are in the strength of the stated conclusions of the evolutionary analyses and some unclear methodological details, which can be fixed with text changes. Overall, this paper will be an important addition to the ciliate MIC/MAC literature and of interest to a broad audience in genomic and evolution.

Major points

#1.1) The horizontal transfer model needs significantly expanded explanation. Are the authors suggesting the horizontal transfers include large segments of DNA or they are ISE/TE-specific. Judging by the shared clade between Pso and Ptr on Figure 2B, the shared ISE family is likely a byproducts of larger scale horizontal transfer between the two species. Given the availability of synteny information based on genome sequencing data, an analysis on whether the horizontal transfer included the flanking regions of FAM_2183 could clarify. The highly interspersed pattern of each species' TEs in figure 2 is mechanistically confusing. The base expectation of one or a few TEs jumping species would be a very different tree.

We have entirely re-written the corresponding section (lines 321-360) in response to several comments of the reviewers (#1.1, #1.12 and #3.7.3). We clarify the points raised here by the reviewer. In brief:

1) We checked synteny conservation for the 1,973 copies inserted in well-conserved coding regions: we only identified 2 copies that are shared by *P. sonneborni* and *P. tredecaurelia*. Thus, the quasi-totality of FAM_2183 copies result from independent waves of insertion in the two lineages, and not from the horizontal transfer of larger genomic fragments (Cf lines 326-332).
2) We agree with the referee: the tree topology on Fig 2B implies that multiple events of horizontal transfer occurred between these two lineages. This observation is corroborated by four other families (FAM_2317, FAM_2321, FAM_2649, FAM_2802) that are shared specifically by *P. tredecaurelia* and the *P. sonneborni/P. sexaurelia* clade. These multiple events of horizontal transfer suggest an important genetic flux between these two lineages. And indeed, we found many large genomic segments (up to 200 kb) that appear to have been introgressed into MIC-specific regions of the *P. sonneborni* genome, from a lineage closely related to *P. tredecaurelia* (these results will be described in detail in another article). The hypothesis that we propose is that these introgressed segments brought some active transposable elements (both autonomous or non-autonomous), including some mobile IESs, which then were able to proliferate within the *P. sonneborni* genome (Cf lines 341-352).

#1.2) The authors should define and clarify their analysis for concluding that there is high sequence conservation and therefore ("beneficial for Paramecium") amongst the member of a repeat family amongst different and distant species. This is not reasonable to conclude so broadly. What defines "highly conserved?" How are we to interpret conservation of a family of repeats? In many other species (and here for fig 5C), specific insertions have been shown to be co-opted, but why or how would a whole family be co-opted? This seems unlikely since most of the insertions should not be beneficial. Alternatively, the authors present some clear and convincing examples of suspected co-option in Fig 5. Without overstating the interpretation of the "conservation" in the 69 subfamilies, the authors could present the data in fig5 as examples that seem likely to be functional, and then state that they also find some other sequences with signatures of conservation which it will be interesting to investigate whether they too could be functional.

We defined ‘highly conserved’ as co-orthologous IESs, conserved across at least 5 of the 8 species of the *aurelia* clade, and showing at least 70% identity between sequences from subclades A and B. We modified our manuscript (lines 454-457) to specify this point. On average, the 69 highly conserved IESs show 87% identity between sequences from subclades A and B. Given the evolutionary distance between these two subclades (average synonymous divergence = 0.95 substitutions/site), such a high level of conservation is clearly indicative of a strong selective pressure. We added a sentence (lines 625-627) to insist on this point, and we added a supplementary Table to report the synonymous divergence between all species of the *aurelia* complex (S6 Table).

We want to emphasize that highly conserved IESs do NOT to correspond to repeated sequences. They are unique sequences (except a few paralogs, notably resulting from the whole genome duplications), and they most probably originated from MAC-destined sequences, and not from transposable elements.

To avoid any misunderstanding, we re-wrote several sections (in the results lines 454-457, 471-475, and in the discussion lines 648-660), to clearly indicate that the origin of these highly conserved IESs differs from that of other IESs (which predominantly derive from transposable elements).

#1.3) The authors frequently use language that puts undue and unnecessary confidence behind interpretation of evolutionary analyses or fail to mention alternative interpretations of their data. For example, the statement (not speculation) that "(line 381 and 572) after their insertion, IESs progressively accumulate changes that make them more efficiently excised" is not supported by the data. Are there sequence motifs in these older IESs that correspond to the higher fidelity excision? Alternatively, the host genome has had more time to adapt to these sequences or a higher fidelity excision mechanism is used for older versus younger IESs. This model would parallel our understanding of KZnFs in mammals.

We agree, the fact that the excision efficiency increases with the age of IESs might be due to changes in cis (within IESs) and/or in trans (in the IES excision machinery). We re-wrote this sentence (lines 392-395):

“This suggests that after a wave of insertions, genomes accumulate changes in cis (within IESs) and/or in trans (in the IES excision machinery) that progressively make these new IESs more efficiently excised, presumably in response to the selective pressure against retention of IESs within coding regions.”

Similarly, in lines 391-394, Ezl1 could be a young mechanism of restriction, older sequences are and have always been restricted with another gene/mechanism.

This scenario is very unlikely because *EZL1* is an ancient gene involved in the elimination of TEs, not only in *Paramecium* but also in *Tetrahymena*.

Minor points:

#1.4 Line 86-91:

Why did the previous analysis of P. tetraurelia find so many fewer copies and families of repeats compared to this approach? Is the current analysis a much improved genome assembly?

The differences between the present study and previous analyses (Arnaiz et al. 2012) are not due to the genome assembly, but to the protocols used to detect copies. In Arnaiz et al. (2012) copies were detected by a simple BLASTN search (all against all comparison of the ~44,000 *P. tetraurelia* IESs). Here we combined data from 9 species and we used HMM profiles, which are more sensitive than BLASTN to detect remote sequence similarities.

But in fact, in terms of number of families of repeats in *P. tetraurelia* , our current results are very similar to those reported by Arnaiz et al. (2012) [we found only one new family, FAM_3, that was not previously reported by Arnaiz et al. (2012)]. And in terms of copy number, we detect about twice as many copies per family (~40 copies in total, if we exclude FAM_3, compared to 23 copies reported by Arnaiz et al.).

FAM_3 corresponds to a family that has been very active at the beginning of the radiation of clade A, but not recently in the *P. tetraurelia* lineage. Hence the 191 FAM_3 copies that we detected in the *P. tetraurelia* genome are quite divergent from each other (which explains why they had not been detected by Arnaiz et al).

Thus, our new analyses are in fact in agreement with the conclusions of Arnaiz et al. (2012): very few recent cases of mobilization of IESs in the *P. tetraurelia* lineage.

We added a sentence in the manuscript (lines 318-320) to clarify this point.

#1.5 Line 95 and discussion:

It would be helpful to elaborate on the model of IESs originating from MAC sequences. As provided, the mechanism by which this could happen is not clear.

We added a paragraph in the discussion on highly conserved IESs to illustrate how IESs can originate from MAC sequences (lines 649-660).

#1.6 Line 122:

The details of the genome sequencing are dense and unnecessary in the text, especially in a broad readership journal like PLOS. Much of this could be moved to supplementary or methods to provide a more accessible and readable text. The first paragraph of the discussion could be used to cover most of the important details of the sequencing.

We have modified the first section of the Results to remove some of the experimental and sequencing details (lines 115-166) and moved that information to the Materials and Methods and supplementary information.

#1.7 Table 1 also seems dense and unnecessarily detailed for the main text.

We simplified Table 1 by moving details on different estimates of genome size to S1 Table.

#1.8 Table 2 is also dense with a lot of information that is not of primary relevance. I would suggest this table contains the key data from the sparse matrix and the rest moved to supplementary (which is a single arrow click away in PLOS). For example, the left half showing repeat-containing stats is secondary to the mobile IES data.

Following suggestions from reviewers 1 and 2, we moved the left half of this table into the supplementary material (S1 Table).

#1.9 Lines 266-270 and other places throughout (lines 498-501):

The authors should take care with their use of the term 'homologous'. For example - "IESs that are homologous, i.e. that result from a single ancestral insertion event". While IES sequences that are found in syntenic loci in two genomes are most likely homologous, so are all the IESs at other loci in these genomes that share significant sequence similarity. It would be better to use orthologous or, as the authors use elsewhere, 'homologous IESs at homologous insertion sites', or invoke synteny.

We agree with the referee: it is important to make the distinction between two categories of homologous IESs:

- homologous IES sequences that result from independent insertion events at different loci
- homologous IES sequences located at homologous insertion sites (which presumably result from a single ancestral insertion event at this site)

We now refer to the first category as ‘*interspersed’* homologs, and to the second category as ‘*co-orthologous*’ IESs. We use the term ‘co-orthologous’ rather than ‘orthologous’, because this category includes paralogs, notably as a result of whole genome duplications.

We now introduced this terminology at the beginning of the Results section (lines 234-240 and lines 263-269) and of the Discussion (lines 537-541), and we use it wherever necessary.

#1.10 Line 324:

"absence of homology" should be "absence of sequence similarity"

Corrected (line 301).

#1.11 Line 339-340:

The text states that FAM_3 is as old as the LCA of subclade A, but then states that "all …IESs that we detected have been subject to recent waves of insertion?"

As shown in Fig 1, we defined ‘recent’ insertions as those that are posterior to the radiation of the *aurelia* clade. To avoid any confusion, we modified this sentence (lines 313-314):

“Thus, all the families of mobile IESs that we detected are relatively recent, posterior to the radiation of the *aurelia* clade.”

#1.12 Line 348-350:

TEs are the exemplar homoplasy-free mutation. The statement that "these two copies correspond to independent insertion events at a same site, rather than ancestral events", needs considerable support. Is there a strong insertion bias of these elements that would limit the potential number of sites? Otherwise, this seems statistically highly unlikely given genome size and number of insertions of this family.

We agree with the reviewer: the finding of two co-orthologous copies is a priori unexpected. We went back to our notebook to check our protocol, and we realized that when we searched for FAM_2183 copies present at orthologous sites, we considered all detected copies, not only those that we classified as ‘mobile IESs’ but also those that are nested within IESs. And it turns out that the two co-orthologous insertion sites that we detected contain nested copies. Thus, one possible explanation is that, by chance, these copies were inserted independently in both lineages within a same pre-existing IES. It is possible that these two co-orthologous copies might have been acquired through the introgression of larger genomic segments (see response to #1.1).

We have entirely re-written the corresponding section (lines 321-360), and we now explicitly discuss the origin of these two co-orthologous copies (Cf lines 352-357) (see also response to point 3.7.3 reviewer 3).

#1.13 Line 384-386:

What was the rational for doing a single knockdowns of Dcl2/3, since the text states that genes require either Ezl alone or both Ezl and Dcl?

Silencing of the *EZL1* gene impairs the excision of 70% of IESs, while co-silencing of the *DCL2/3* genes impairs the excision of 7% of IESs, all among the of *EZL1*-dependent IESs. To clarify, we rephrased the sentence (lines 397-400).

#1.14 Lines 448-45:

"Among the 56 families of highly conserved IESs present in P. tetraurelia, 10 (18%) are transcribed at substantial levels (>1 RPKM) during autogamy (as compared to 0 0.8% for other IESs) (S4 Table)." Wouldn't we expect that less diverged, younger sequences would be more likely to retain promoter activity and therefore be transcribed, without need to invoke co-option?

Highly conserved IESs are not young (they predate the divergence of the *aurelia* clade), and they do not derive from transposable elements (see response to #1.2). So the fact that they are transcribed cannot be attributed to an ancestral promoter activity associated to transposable elements.

#1.15 Line 516:

"must correspond to non-autonomous elements" should be "likely correspond…"

Corrected.

#1.16 Lines 590-596:

Are the genes required for excision near the insertion sites of these "conserved" families of IESs? This seems highly speculative and should be rephrased to reflect this.

In the corresponding section of the discussion we wrote “it is tempting to speculate that these IESs may play a role in controlling the IES excision machinery itself” (lines 637-638). So we think we made it clear that we are here developing a working hypothesis.

#1.17 Lines 719-720:

"the separation of somatic and germline functions between the MIC and the MAC offered the possibility for selfish genetic elements to invade genes in ciliates." Isn't it equally or more likely that the invasion of TEs drove the complex biology of separating germ/soma via MIC/MAC?

The reason why ciliates have evolved this complex MIC/MAC genetic system is a matter of debate. At first sight, one benefit of the MIC/MAC separation is that it allows cells to get rid of repeated sequences during the development of the somatic genome, and thereby to avoid their deleterious phenotypic impacts. Thus, as mentioned by the reviewer, one possible hypothesis is that the mutational pressure due to TE insertions might have driven the evolution of the MIC/MAC separation. This ‘TE-driver’ hypothesis raises however several major issues. First, with this model, it is difficult to see what could be the intermediate evolutionary steps: the benefits, in terms of mitigation of damages caused by TE insertions, would not appear before the evolution of a fully functional genetic system (MIC/MAC separation + efficient TE excision machinery). So, this model raises the question of the early benefits that initiated the evolution of this MIC/MAC separation. Second, this TE-driver model also raises an issue of temporal logic. Indeed, this benefit of the MIC/MAC separation *first* requires the evolution of an efficient TE excision machinery (as long as TE excision is not efficient, what is the benefit of having a MAC?). Hence, this hypothesis implies that the efficient TE excision machinery evolved *before* the MIC/MAC separation. But then, if an organism already has a machinery to excise TEs from its genome, what is the benefit of separating MIC/MAC?

Of course, we know that the arms race between intragenomic parasites and their hosts can sometimes lead to very unexpected evolutionary scenarios. We therefore agree with the referee that it is *a priori* tempting to speculate that TEs might have played a role in the evolution of the MIC/MAC genetic system. However, beyond this intuition, when one tries to formulate more precisely this hypothesis, then one meets with the difficulties mentioned above.

This is why we consider much more parsimonious a scenario where the MIC/MAC separation would have evolved first, for another reason (e.g. the high ploidy of the MAC nucleus might be beneficial to sustain very large cells (see Cheng et al 2020), and thereby created a new niche for selfish genetic elements.

Cheng et al 2020. Curr Biol. 2020;30: R502–R510. doi:10.1016/j.cub.2020.02.026

# Reviewer #2:

This is a well-written and well-argued manuscript that focuses on the evolutionary dynamics of germline-limited sequences (i.e. IESs) in multiple species of the genus Paramecium. The authors use a comparative approach that combines high-throughput sequencing of isolated germline micronuclei (and occasionally somatic macronuclei) with a suite of bioinformatic tools/approaches. Most importantly, the analyses and resulting insights on IES dynamics are well-justified, and the manuscript is generally written in a manner that will make the complex biology of ciliates, plus the interest results here, accessible to broad PLOS Biology readership.

MAJOR CONCERNS

#2.1- I am confused by the claim that thousands of IESs were acquired by horizontal transfer… are these really thousands of HGTs or a smaller number coupled with intragenomic expansions (lines 108, 542-545).

We agree with the reviewer that this sentence was misleading. What we show is that 1) several families of mobile IES generated thousands of recent insertions and 2) some of these families have been subject to horizontal transfer (but most of the copies that we detected result from intragenomic expansions, posterior to the transfer events).

We re-wrote to avoid confusion (lines 542-545: now lines 591-592)(ex line 108 at the end of the introduction has been removed, lines 102-105). We also have entirely re-written the result section describing the scenario of proliferation of mobile IESs after horizontal transfer (lines 321-360).

#2.2- There is perhaps a bit too much conclusion/discussion in the intro… and missing is a brief intro to the murkiness/history of species designations in the genus Paramecium. Similarly, parts of the discussion is a bit overwritten (see below) and perhaps some of the methods could be move to the supplement, though this would be an editorial decision.

To address this comment, we essentially removed the last paragraph of the introduction (lines 102-105), which was redundant with the abstract.

We now refer to an excellent, detailed and up-to-date description of the *aurelia* group of species for the reader interested in the complex history of species designation in *Paramecium* (line 102).

We re-organized the end of the discussion, which is now subdivided in two sections. The last section “Parallel scenario for the evolution of IESs and spliceosomal introns” is shortened (lines 748-780).

#2.3- The final section of the discussion, on IESs and spliceosomal introns, seems overwritten given the data. For example, the claim that the "the nuclear envelope opened the way for introns to invade genes' requires both citation (see, for example, papers by William Martin and Eugene Koonin) and a more nuanced interpretation. I suggest the authors tighten substantially here to better reflect the data in this manuscript - at least some of this long speculative section might be better in a subsequent review paper.

We agree that the final section of the discussion is speculative. We have reorganized the last part of the discussion in two distinct sections and have shortened the paragraph regarding the parallel between IES and intron evolution (lines 748-780).

#2.4- The sequence logos and phylogenetic analyses (Fig 2) are not particularly compelling. For one, the first sequences seems to reflect just the compositional bias (i.e. AT richness) so here, reanalyzing using shuffled sequences AND reporting on these data is critical.

To take into account the high AT-content of IESs, the information content reported in the sequence logo was computed relative to the observed base composition of IES (we modified the legend of Fig 2 to indicate this point; lines 374-375). As already mentioned in the manuscript, all copies show a high level of sequence similarity (average pairwise identity between randomly sampled copies = 72%). The reviewer could have a look at the multiple alignment of this family (as indicated in the Data Availability section, multiple alignments of mobile IES are available on Zenodo: https://doi.org/10.5281/zenodo.4726682). There is no doubt that the strong sequence similarity between all these IESs reflects homology and not just a compositional bias.

#2.5 Also, what is the length variation for Fam_2183… are the all 233bp with no variance?

The consensus sequence is 233 pb-long, and a large number of copies have this exact size. But of course, there are also many copies that have accumulated deletions. We now display the length distribution in Fig 2C.

#2.6 Similarly, the 'phylogeny' is suspect as rapid rates of evolution may make alignment/homology assessment very difficult here. In other words, the star-like shape could be due to either rapid burst OR (as discussed elsewhere in the manuscript) rapid rates of evolution obscuring homolog assessment. I would revisit effect of compositional bias (for motifs) and homology assessment (for tree), and then perhaps move this part to the supplement.

We disagree: with an average pairwise identity of 72% among all members of the family, sequences are very far from saturation of the phylogenetic signal. Given the high level of sequences similarity, and the quite narrow length distribution of copies, the multiple alignment is in fact relatively easy, with little ambiguity. We see no reason why this phylogenetic tree should not be considered as reliable.

MINOR CONCERNS:

#2.7- Line 64: word choice… not really 'destroyed' but instead recycled?

The maternal MAC is fragmented into approximately 30 pieces at the end of MIC meiosis. These fragments, in which DNA replication is stopped, are lost by dilution once cell divisions resume. We and others commonly use the term “destroyed” to describe the series of events leading to the disappearance of the maternal MAC. We note that in another ciliate, *Tetrahymena*, the old MAC undergoes an apoptosis-like process and is recycled. There is no evidence for such a pathway in *Paramecium*.

#2.8- Measure of 'substantial burden' (line 110) might best be done with fecundity studies… also, there is a bit too much conclusion in the section of the intro. Please rewrite/rethink.

We have considerably shortened the last part of the introduction.

#2.9- Similarly, the section entitle 'fitness consequences' (line 555) might be better toned down/tightened… no fitness is measured here, just inferred. So perhaps 'Possible fitness consequences…'

We renamed this section (line 603):

‘Indirect evidence of mutational burden caused by IES invasions’

#2.10- A bit more discussion of MIC genome size and % of retention in MAC genomes might be warranted here, or in a subsequent manuscript.

We dedicated a paragraph at the end of the first Results section and another one at the beginning of the Discussion to comment on MIC and MAC genome sizes with what seems to us the most important information regarding this matter.

#2.11 Similarly, more discussion of relationship between IES presence and gene expression data would also be nice. These are such impressive comparative data.

In our initial paper on the IES repertoire in *P. tetraurelia*, we already had described the relationships between IES presence and gene expression (Arnaiz et al. 2012). We agree with the referee that it is nice to confirm this pattern with 8 additional *Paramecium* species. However, on this specific point, there is not much more to say than what we already had discussed in Arnaiz et al. 2012. This is why we present this result in the supplementary material (S7 Fig) and we do not push the discussion of this result much further (lines 203-207).

#2.12- The periodicity of IES lengths might be worth some additional attention.

This point has already been discussed in detail in (Arnaiz et al. 2012). We rephrased the sentence to make it clear: “As previously observed for P*. tetraurelia* (Arnaiz, 2012), IESs have a characteristic length distribution…”(lines 420-423).

#2.13- The logic of the section on "why IESs are not eliminated" (598…) feels a bit tautological/circular, and the too-long paragraph here makes this section a bit difficult to follow. Please rethink/refine, and perhaps consider the concept of evolutionary constraint. Perhaps some/many IESs are functionally neutral, which may be consistent with the observation of loss of IESs from highly expressed genes? I am suggesting rewording and shortening here.

We shortened this section, and we now simply focus on the discussion of the mechanisms of IES loss (lines 662-682).

#2.14- Table 2 is a bit difficult to interpret as presented… especially given the discussion of TEs that follows. I would encourage the authors to rethink there, perhaps synthesizing the data a bit more to explicitly link to TE families and moving this detailed version to supplemental materials

Following suggestions from reviewers 1 and 2, we moved the left half of this table into the supplementary material (S1 Table).

#2.15- From figure 1 legend: the claim that estimates of loss rate along terminal branches … may be overestimated is true, but doesn't the logic also go the other way - that some IESs may have been gained and lost, and therefore undetected…

The model that we used does take into consideration the possibility that multiple events occurred along a given branch of the phylogeny. Thus, as long as the phylogenetic signal is not saturated (which is not the case here), it is possible to estimate true gain/loss rates, even with some unobservable events.

#2.16- Figure 3 is very cool, but not accessible to general reader with current legend/titles.

We modified the legend and title to make it more accessible (lines 409-418).

#2.17- I wonder if there is a way to add an inset to Fig S14, or at least to point to it in legend, of Figure 1

We modified the legend of Fig 1 to point to S8 Fig (ex S14 Fig).

# Reviewer #3:

Summary:

In this report Sellis et al. studied multiple Paramecium species and examined the evolutionary history of IES gain throughout the clade. The authors identified IES sequences by assembling the macronuclear genomes of these species and examining inserted sequences from micronuclear reads when compared to the MAC assemblies. Once each species' repertoire of IESs had been identified, the authors inferred the relative age of IESs by examining the presence or absence of certain IESs between the different species in comparison with their phylogenetic distribution. The authors found that older IESs are more easily excised from the genome during rearrangement, requiring fewer excision pathways to be successfully removed. They discovered multiple families of IESs that show evidence of recent acquisition and expansion, with certain families having hundreds to thousands of copies present within different species, some of which share homology with known transposable elements. Their

results provide evidence that IESs are derived from degenerated transposable elements, which has long been hypothesized to be the case in the field. In addition, they identify a set of IESs encoding a protein under strong purifying selection, suggesting that certain IESs have a beneficial effect on the fitness of these species through either encoding proteins or regulating gene expression. The work in this manuscript is sound, but there are some minor issues that should be addressed before it is published.

Specific comments:

#3.1. The main text has an abundance of specific numbers scattered throughout that can already be found in the tables. Removing some of these numbers and referencing the tables in the text instead would improve the readability of some sections for a more general audience.

We have tried our best to address this point by removing some of the numbers at different places in the main text. Yet, we kept the numbers when it seems important to us to convey the message.

#3.2. While the data presented in this report is generally robust, it suffers from a lack of statistical testing. Adding significance testing would strengthen some of the conclusions, such as in figures 3 or S9.

We now present the results of statistical tests in Fig 3, S10 and S12 (all the results we reported are supported by these tests).

#3.3. In the Results section on page 7, the portion of text describing the methods used for genome size estimation should be moved to Methods.

We have done as suggested (lines 115-142).

#3.4. Since the sequencing depth of the MIC genomes has a significant impact on the identification of IESs, details of the MIC genome coverage should be included in Table 1.

As suggested, new Table 1 now includes the sequencing depth of the MIC genomes. We also modified the table to add the estimated number of IESs for pson (in parenthesis).

#3.5. The high proportion of repeat content in the P. caudatum MIC is curious. Could this be an artifact of overamplified reads in the sequencing library? Adding another method of repeat content estimation, such as some long-read Nanopore or PacBio sequencing, could help reinforce this claim, if such data are easily generated or already available.

It is true that amplification biases during the preparation of Illumina sequencing libraries may cause some variation in sequencing depth along the genome. However, such variation is relatively limited (e.g. up to 4-fold variation in sequencing depth between GC-rich and GC-poor DNA fragments; Benjamini & Speed 2012). Here, the two satellite repeats (respectively 332 bp and 449 bp) account for 71% of all MIC reads (i.e. equivalent to a sequencing depth of 15Mx on these two repeats, compared to 13x on the entire genome). It seems totally unlikely that amplifications artefacts could cause this 1 million-fold variation in sequencing depth.

Note also that the presence of these repeats was confirmed by PCR amplification from whole genomic DNA in the strain that was sequenced, as well as in several other strains of *P. caudatum* (S4 Fig).

Reference: Benjamini Y and Speed TP 2012 Summarizing and correcting the GC content bias in high-throughput sequencing. Nucleic Acids Research 40: e72–e72. https://doi.org/10.1093/nar/gks001

#3.6. Many MAC genome assemblies were assembled for this study, and the IES detection process is dependent on the quality of the assembled genomes. Some measurements of genome completeness, such as BUSCO scores or tRNA representation, would be a good addition, and are usually used to assess completeness of other ciliates MAC genomes..

We performed a BUSCO assessment, which confirmed the good completeness of MAC genomes assemblies, with only 0.6% to 1.8% of missing BUSCO genes. Details on BUSCO results are now included in S2 Table.

#3.7. It is claimed that the presence of certain families in both P. sonneborni and P. tredecaurelia is suggestive of horizontal transfer. Do the geographic distributions of these species overlap?

Most of the species of the *aurelia* complex are cosmopolitan (Przyboś & Tarcz. 2018). *P. tredecaurelia* and *P. sonneborni* are rare species, but they have been reported at very distant places around the world: France, Mexico, Madagascar, Thailand and Israel for *P. tredecaurelia* (Przyboś et al. 2013); USA and Cyprus for *P. sonneborni* (Przyboś et al. 2015). Thus, the geographic distribution of these species is very wide. We added a sentence to mention this point (lines 357-358).

Przyboś et al. 2013. Acta Protozool. 52:257-266. doi: 10.4467/16890027AP.13.022.1314.

Przyboś and Tarcz. 2018. Folia Biol. (Kraków) 66:111-119. doi: 10.3409/fb_66-3.12.

Przyboś et al. 2015. Protist 166:438-456. doi: 10.1016/j.protis.2015.07.001.

#3.7.2 Do they share any food sources or symbionts that could have been involved in this transfer?

In the lab, all species are fed with the same bacteria, but little is known regarding the specific ecology of each *aurelia* species in nature.

#3.7.3 Can a scenario of shared ancestry followed multiple losses/degeneration in all the other lineages be entirely ruled out?

We identified 5 families of mobile IESs showing the same patchy phylogenetic distribution, suggestive of horizontal transfers between *P. sonneborni* and *P. tredecaurelia* lineages (FAM_2183, FAM_2317, FAM_2321, FAM_2649, FAM_2802). The alternative hypothesis of vertical inheritance of the families since the last common ancestor of the *aurelia* complex would imply that all copies of each family have been lost independently in three lineages of subclade A, which is a priori very unlikely. Most importantly, the phylogeny of the FAM_2183 family is mainly star-like, with *P. sonneborni* and *P. tredecaurelia* copies interspersed across the entire tree (Fig 2B). This topology is incompatible with a scenario of vertical inheritance, in which copies from each species would have been expected to form two distant clades.

We have entirely re-written the corresponding section (lines 321-360) to discuss these points (and also in response to points 1.1 and 1.12 of reviewer 1).

#3.7.4 Are some IES families not detected because of low MIC sequencing depth?

We performed an analysis of the sensitivity of IES detection according to MIC sequencing depth (see below). This analysis showed that for most species, the sequencing depth was sufficient to detect >99% of IESs. The only exceptions are *P. caudatum*, and *P. sonneborni*, where the MIC sequencing depth was limited. Thus, the fact that some mobile IESs (FAM_2183, FAM_2317, FAM_2321, FAM_2649, FAM_2802) are shared by *P. sonneborni* and *P. tredecaurelia* but absent in other *aurelia* species cannot be explained by a limited sensitivity of IES detection in these species.

#3.8. A scalebar would be nice for the tree on the right of Fig. 3.

This tree is not drawn to scale (see the dash bars on the *P. caudatum* branch). This tree is displayed only to identify the speciation nodes to which we refer in the left panel. The complete phylogenetic tree (with a scalebar) is given in Fig 1.

#3.9. For Figure 4A, the low coverage and high repeat content of the sequencing for P. caudatum makes me skeptical of its chart. Given the disparity in the amount of data between the clades, the comparison between them does not seem too meaningful. The chart of the aurelia clade alone would be fine.

The high repeat content in *P. caudatum* is not *per se* an issue for IES detection (sequence reads from the satellite repeats do not map on the MAC genome assembly, and hence do not interfere with the process of IES detection). However, because of the huge MIC genome size (caused by these repeats), the sequencing depth was low, which limited the sensitivity of IES detection in *P. caudatum*.

The concern of the reviewer is that this artefact might also have biased the length distribution of detected IESs (Fig 4A). To assess this point, we performed an experiment: we subsampled sequence reads from the *P. tetraurelia* MIC dataset so that to obtain subsets of lower depth (from 2x to 30x), on which we applied the same IES detection procedure. We then compared IESs detected on each subset to those identified in the entire dataset. As expected, the sensitivity of IES detection decreases when sequencing depth is low (<15x). However, the length distribution remains unchanged, whatever the sequencing depth. In other words, the sequencing depth affect the sensitivity of IES detection for all IESs, independently of their length. We therefore conclude that the difference in IES length distribution between *P. caudatum* and *P. aurelia* species (Fig 4A) is not an artifact due the low sequencing depth in *P. caudatum*.

We included this new analysis in the revised manuscript (lines 180-181, and supplementary S5 Fig).

#3.10. Figure S7B does not have a label on its Y-axis.

Corrected.

#3.11 Intergenic IESs will be harder to distinguish because they atrophy faster, so there will always be a bias towards annotating IESs in coding regions that interrupt MDSs, over intergenic background.

This is incorrect: IESs are detected in each species by mapping MIC sequence reads on the corresponding MAC genome assembly. The detectability of IESs therefore does not depend on their evolutionary rate, nor on the evolutionary rate of the locus in which they are inserted (coding regions or intergenic). It only depends on the MIC sequencing depth (which is the same in coding or non-coding regions).
