# Supplementary material for: Massive colonization of protein-coding exons by selfish genetic elements in Paramecium germline genomes
Source: PLoS Biol. 2021 Jul 29;19(7):e3001309. doi: 10.1371/journal.pbio.3001309 (PMC8354472; doi:10.1371/journal.pbio.3001309)
Supplement: Attachment — Submitted filename: Cover_letter_revision_2.docx [file pbio.3001309.s024.docx]

Dr. Roland G Roberts	Paris, May 31, 2021

Editor

PLOS Biology

Dear Dr. Roberts,

We are now submitting a revised version of our manuscript PBIOLOGY-D-21-00087R2 entitled "Massive colonization of protein-coding exons by selfish genetic elements in *Paramecium* germline genomes”.

As requested, we checked that the individual numerical values for Figs 1, 2AC, 3, 4AB, S2, S3, S4A, S5ABCDEFGH, S7AB, S10AB, S11, S12AB were provided. The data underlying each cited Figure/Panel may be found in https://doi.org/10.5281/zenodo.4836464.

This sentence was added in the Data availability section and in each relevant Figure legend.

We provide a file where the changes are highlighted in the manuscript text.

Thank you for considering our revised manuscript.

If you have any questions, please do not hesitate to contact us.

With best regards,

Sandra Duharcourt

Signed on behalf of all the authors.
